# The mitochondrial genome of *Diostracus lamellatus* (Diptera: Dolichopodidae)

**DOI:** 10.1080/23802359.2018.1450662

**Published:** 2018-03-14

**Authors:** Qilemoge, Shang Gao, Chufei Tang, Ning Wang, Ding Yang

**Affiliations:** a College of Plant Protection, China Agricultural University, Beijing, China;; b Institute of Grassland Research, Chinese Academy of Agricultural Sciences, Hohhot, China

**Keywords:** Mitochondrial genome, Hydrophorinae, phylogenetics

## Abstract

The long-legged fly *Diostracus lamellatus* Wei et Liu belongs to the subfamily Hydrophorinae of Dolichopodidae. The mitogenome of *D. lamellatus* was sequenced, the first representative of the mitogenome of the subfamily. The mitogenome is 14,143 bp totally, consisting of 13 protein-coding genes, two rRNAs, and 22 transfer RNAs. All genes have the similar locations and strands with that of other published species of Dolichopodidae. The nucleotide composition biases towards A and T, which together made up 75.8% of the entirety. Bayesian inference analysis strongly supported the monophyly of Dolichopodidae. It suggested that the Hydrophorinae is the sister group to the clade of Sciapodinae + Dolichopodinae.

Hydrophorinae is a large subfamily of the family Dolichopodidae, the genus *Diostracus* is the third largest genus of the subfamily Hydrophorinae with 100 species known in the world (Yang et al. 2006, 2011; Grichanov 2017). Primarily distributed in the Holarctic and Oriental regions, species of *Diostracus* are usually found in the habitats near freshwater on the stones and plants in or near the streams. Their diversity is used as an index of evaluating the quality of aquatic environment.

The male adult specimens of *D. lamellatus* used for this study were collected from Mount Taibai (33°51′141″N, 107°50′328″E, 1781 m) of Shaanxi Province in China in 2015. The specimens of *D. lamellatus* were deposited in the Entomological Museum of China Agricultural University (CAU). The total genomic DNA was extracted from the whole body (except head) of the specimen using the QIAamp DNA Blood Mini Kit (Qiagen, Hilden, Germany) and stored at –20 °C until needed. The mitogenome was amplified and sequenced as described in our previous studies (Wang, Li, et al. 2016). The nearly complete mitogenome of *D. lamellatus* is 14,143 bp. It encoded 13 PCGs, 22 tRNA genes, two rRNA genes and the control region could not be sequenced entirely in this study, and were similar with related reports before (Kang et al. 2014; Li et al. 2016; Wang, Ding, et al. 2016; Wang, Liu, et al. 2016; Wang, Wang, et al. 2016; Li et al. 2017; Zhou et al. 2017). The nucleotide composition of the mitogenome was biased towards A and T, with 75.8% of A + T content (A = 38.5%, T = 37.3%, C = 14.4%, and G = 9.9%). The A + T content of PCGs, tRNAs, and rRNAs is 74.8%, 77.5%, and 80.9%, respectively. The total length of all 13 PCGs of *D. lamellatus* is 11,218 bp. All PCGs in *D. lamellatus* utilize the conventional translational start codons for invertebrate mtDNA. For example, five PCGs (*ATP8*, *ND1*, *ND2*, *ND3*, and *ND6*) initiated ATT codons, and six PCGs (*COII*, *COIII*, *ATP6*, *ND4*, *ND4L*, and *CYTB*) initiated with ATG codons, *COI* and *ND3* initiated with ATC as a start codon. Eleven PCGs used the typical termination codons TAA and two PCGs (*CYTB*, *ND3*) used TAG in *D. lamellatus*.

Phylogenetic analysis was performed based on the nucleotide sequences of 13 PCGs from 11 Diptera species. Bayesian (BI) analysis (Figure 1) showed that monophyletic Empidoidea was assigned to be the sister group to the clade of Xylomyidae, Xylophagidae, and Asilidae. Empididae and Dolichopodidae, monophyletic Empididae was assigned to the sister to monophyletic Dolichopodidae. For the phylogeny of Dolichopodidae, Neurigoninae was assigned to be the sister of the clade of Hydrophorinae, Sciapodinae, and Dolichopodinae. The phylogenetic relationship within Empidoidea is very clear: Empididae + (Neurophorinae + (Hydrophorinae + (Sciapodinae + Dolichopodinae))). The position of Hydrophorinae was also supported by the morphological study (Yang et al. 2006, 2011). The mitogenome of *D. lamellatus* could provide the important information for the further studies of Dolichopodidae phylogeny.

**Figure 1.**
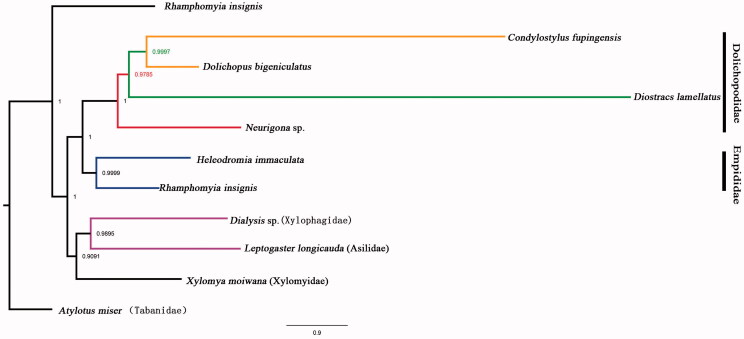
Bayesian phylogenetic tree of 11 Diptera species. The posterior probabilities are labelled at each node.
